# Linac‐based stereotactic radiosurgery (SRS) in the treatment of refractory trigeminal neuralgia: Detailed description of SRS procedure and reported clinical outcomes

**DOI:** 10.1002/acm2.12057

**Published:** 2017-02-28

**Authors:** Damodar Pokhrel, Sumit Sood, Christopher McClinton, Habeeb Saleh, Rajeev Badkul, Hongyu Jiang, Timothy Stepp, Paul Camarata, Fen Wang

**Affiliations:** ^1^ Department of Radiation Oncology The University of Kansas Cancer Center Kansas City KS USA; ^2^ Department of Neurosurgery The University of Kansas Cancer Center Kansas City KS USA

**Keywords:** linac‐based SRS, neuralgia, outcome, toxicity, trigeminal

## Abstract

**Purpose/Objectives:**

To present our linac‐based SRS procedural technique for medically and/or surgically refractory trigeminal neuralgia (TN) treatment and simultaneously report our clinical outcomes.

**Materials and Methods:**

Twenty‐seven refractory TN patients who were treated with a single fraction of 80 Gy to TN. Treatment delivery was performed with a 4 mm cone size using 7‐arc arrangement with differential‐weighting for Novalis‐TX with six MV‐SRS (1000 MU/min) beam and minimized dose to the brainstem. Before each treatment, Winston–Lutz quality assurance (QA) with submillimeter accuracy was performed. Clinical treatment response was evaluated using Barrow Neurological Institute (BNI) pain intensity score, rated from I to V.

**Results:**

Out of 27 patients, 22 (81%) and 5 (19%) suffered from *typical* and *atypical* TN, respectively, and had median follow‐up interval of 12.5 months (ranged: 1–53 months). For 80 Gy prescriptions, delivered total average MU was 19440 ± 611. Average beam‐on‐time was 19.4 ± 0.6 min. Maximum dose and dose to 0.5 cc of brainstem were 13.4 ± 2.1 Gy (ranged: 8.4–15.9 Gy) and 3.6 ± 0.4 Gy (ranged: 3.0–4.9 Gy), respectively. With a median follow‐up of 12.5 months (ranged: 1–45 months) in *typical* TN patients, the proportion of patients achieving overall pain relief was 82%, of which half achieved a complete pain relief with BNI score of I‐II and half demonstrated partial pain reduction with BNI score of IIIA‐IIIB. Four *typical* TN patients (18%) had no response to radiosurgery treatment. Of the patients who responded to treatment, actuarial pain recurrence free survival rates were approximately 100%, 75%, and 50% at 12 months, 15 months, and 24 months, respectively. Five *atypical* TN patients were included, who did not respond to treatment (BNI score: IV–V). However, no radiation‐induced cranial‐toxicity was observed in all patients treated.

**Conclusion:**

Linac‐based SRS for medically and/or surgically refractory TN is a fast, effective, and safe treatment option for patients with *typical* TN who had excellent response rates. Patients, who achieve response to treatment, often have durable response rates with moderate actuarial pain recurrence free survival. Longer follow‐up interval is anticipated to confirm our clinical observations.

## 1 Introduction

Trigeminal neuralgia (TN) is a neurologic syndrome that presents with spontaneous episodes sever, electric shock‐like pain along the trigeminal nerve dermatome(s). Typical primary treatment strategies consist of medical management with antiseizure medication, surgical intervention such as microvascular decompression, and stereotactic radiosurgery.^1^, ^2^, ^3^, ^4^ Historically, gamma knife‐based stereotactic radiosurgery (SRS) has been considered an effective and noninvasive alternative treatment modality associated with minimal toxicity — particularly in patients with medically and surgically refractory TN or those who are not ideal surgical candidates.^5^, ^6^, ^7^, ^8^, ^9^ For example, in a multi‐intuitional review of 503 patients with TN who had been treated with gamma knife‐based SRS, 58% of patients achieved complete pain relief and 36% of patients achieved partial pain relief.^8^

Linac‐based SRS has become an increasingly popular treatment modality for TN due to technological advancements which have allowed for precise radiation delivery in a fast and effective manner.^10^, ^11^, ^12^, ^13^Recently, many researchers have presented linac‐based SRS treatment outcomes for TN patients which are comparable with gamma knife data.^14^, ^15^, ^16^, ^17^, ^18^, ^19^ Due to the effectiveness of linac‐based SRS for treatment of smaller target such as TN, we sought to present a detailed description of our linac‐based SRS technique as well as report our long‐term clinical outcomes in patients with medically and/or surgical refractory TN.

## 2 Materials and methods

### 2.A Patient imaging and frame placement

After obtaining approval from our institutional review board, a retrospective review was conducted consisting of a total of 27 TN patients who had been treated at our institution from 2009 to 2016 using frame‐based, linac‐based SRS. All patients underwent a high‐resolution magnetic resonance imaging (MRI) scan consisting of 1 mm thin slices with T1‐weighted, T2‐weighted, and 3D‐fast imaging employing steady state acquisition (FIESTA) sequences prior to treatment. On the day of radiosurgery treatment, an experienced neurosurgeon placed a BrainLAB stereotactic frame on the patient's head after application of a local anesthetic. Depth Helmet bobble^20^ measurement was performed for quality assurance of the frame placement and, immediately thereafter, the patient was set up for the planning computerized tomography (CT) simulation which was performed on a 16 slice Phillips Brilliance Big Bore CT Scanner (Phillips, Cleveland, OH) and BrainLAB CT localizer (BrainLab Head&Neck Localization Inc., Heimstetten, Germany). CT simulation images were acquired with 512 × 512 pixels at 0.75 mm slice thickness and 0.75 mm slice spacing following departmental SRS scanning protocol.

### 2.B Target delineation and SRS treatment planning

The MRI was co‐registered with the planning CT image set and an experienced neurosurgeon and radiation oncologist delineated the trigeminal nerve root (TNR), for isocenter placement, using the 3D‐FIESTA MRI sequence. The target was localized to the base of the trigeminal nerve at the junction of nerve entry into Meckel's Cave and exit from the brainstem. Organs at risk (OAR) were delineated on the co‐registered MRI and consisted of the following structures: brainstem, optic apparatus (optic chiasm and bilateral optic nerves), eyes and lenses, and temporal lobe of the brain.

**Figure 1:**
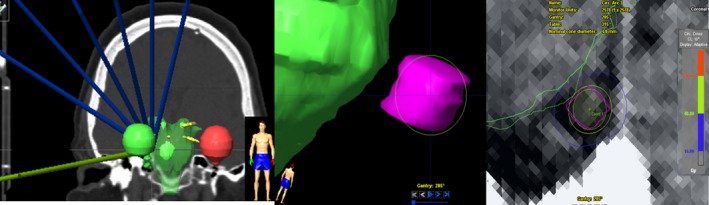
Left: example seven‐arc arrangement (frontal view) for the treatment of right‐sided TN. Middle: corresponding DRR clearly showing a 4 mm diameter cone encompassing 3D‐view of TNR (pink) and proximity of the brainstem (green). Right: resulting field of view of 4 mm diameter cone (green) and associated IDLs for 40 Gy (dark yellow) and 16 Gy (blue).

For each treatment, a seven‐arc plan was devised in iPlan BrainLAB to deliver the single‐fraction prescription dose to the 100% isodose line (IDL), using six MV‐SRS beams (1000 MU/min), and a 4 mm diameter cone size. The treatment plans were optimized in order to minimize brainstem dose as well as avoided beam entry through the eyes. All treatment plans were performed using heterogeneity corrected pencil‐beam algorithm with 1.0 × 1.0 × 1.0 mm^3^ grid sizes for dose calculations. All plans employed a single‐fraction point dose of 80 Gy to the TNR and were forward‐optimized to maintain a maximum TNR point dose of 80 Gy, 40 Gy (50%, IDL) encompassing the TNR diameter, and maximum brainstem point dose less than 16 Gy. One example patient case (right trigeminal patient) of seven‐arc arrangement with associated digitally reconstructed radiograph (DRR) is shown in Fig. 1. In general, the total average arcing length of 130° (e.g., for right trigeminal nerve, 200 to 330°, clockwise rotation for each arc) was used and the couch separation was chosen from 15 to 35°. Due to the use of orbital avoidance vertex‐arc arrangement, the elliptical dose distribution along the longitudinal direction of TNR (optimized for target coverage) was devised that also reduced dose to brainstem and optic apparatus.

### 2.C Evaluation of dose distribution

**Figure 2:**
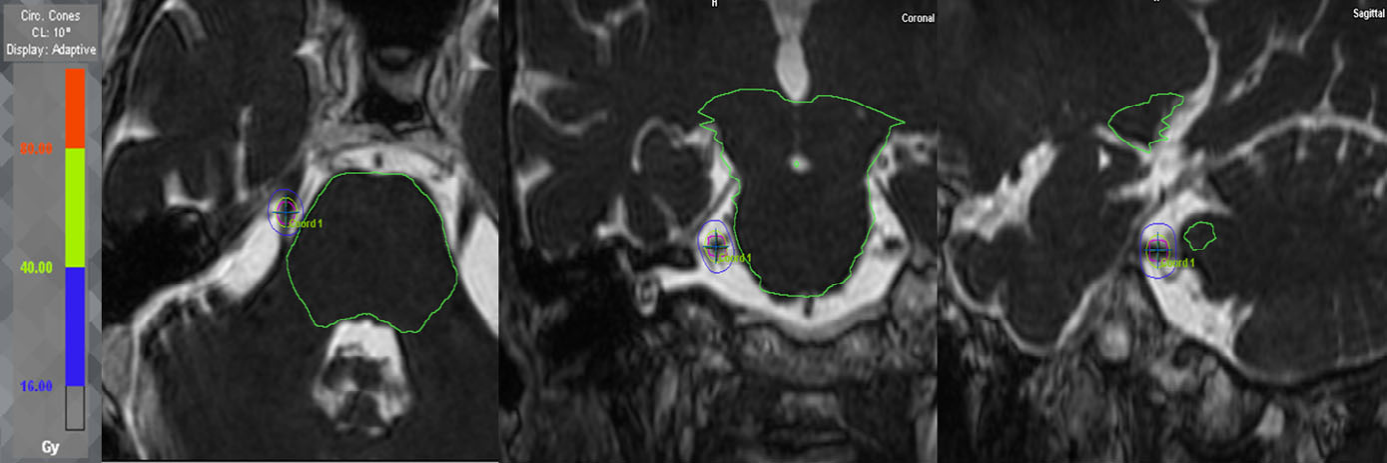
Dose distribution for a 62‐yr‐old male with refractory right trigeminal neuralgia. An 80 Gy point dose to the isocenter was prescribed. The IDLs for 40 Gy (light green) and 16 Gy (blue) are clearly shown in conjunction with contours for brainstem (green) and TNR (red). The isocenter was localized by identifying the midpoint between the trigeminal eminence where the dorsal root merges with the lateral pons (brainstem) and entry into Meckel's cave (see plus sign – Coord 1 in all 3‐view). A 4 mm diameter circular cone and seven noncoplanar differentially weighted arcs were used to minimize brainstem dose. A total of 19,140 MU was delivered with a total beam‐on‐time of 19.14 min (not including couch kick time). In this particular case, max‐dose to brainstem was 14.9 Gy, dose to 0.5 cc of brainstem was 3.8 Gy, max‐dose to optic apparatus was less than 1.5 Gy, and max‐dose to eyes and lenses were 0.6 Gy and 0.1 Gy, respectively. Follow‐up at 13 months demonstrated that this patient had achieved complete pain relief (no pain, no medication, and BNI score of I).

**Figure 3:**
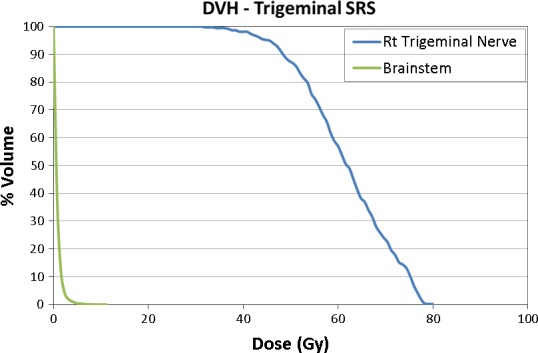
Representative DVH for the same example patient demonstrating a TNR maximum point dose of 80 Gy, TN nerve 40 Gy volume of about 100%, maximum brainstem point dose of 14.9 Gy, and dose to 0.5 cc of brainstem was 3.8 Gy. Considering the voxel size effect, dose calculation inaccuracy, and contouring irregularity, nearly 40 Gy (50% IDL) covered 100% of the contoured TN.

For all TN SRS treatment plans, a dose–volume histogram (DVH) was generated in the iPlan BrainLAB TPS and subsequently evaluated by an experienced radiation oncologist, neurosurgeon, and medical physicist to ensure acceptable OAR doses were achieved. In addition to maximum dose to brainstem, the dose to 0.5 cc of brainstem was also documented. Dose distributions for an example patient are shown in Fig. 2 and the corresponding DVH is shown in Fig. 3.

### 2.D Independent second MU check

A most commonly used TMR‐based spreadsheet independent MU calculation was devised and clinically implemented for second MU check. An independent MU verification is mandatory for safe and effective delivery of such a complex treatment plan. For the given SRS beam, the TMR‐based spreadsheet calculation takes into account of the 4 mm cone size output factors and independently computes MU on the arc‐by‐arc basis for the approved TN SRS treatment plan. For all patients, on a per‐arc basis, our computed BrainLab iPlan MU matched with TMR‐based spreadsheet calculation within ± 3.0%.

### 2.E Machine quality assurance and patient setup

For the given collimator, couch, and gantry rotations, daily Winston–Lutz (WL) QA tests^10^ were performed using a 7.5 mm circular cone and a couch mount with a 5 mm diameter mechanical bearing ball (BB). In our clinic, due to the integration of WL QA procedure with ExacTrac system, ExacTrac system was calibrated before the WL QA with a pair of oblique kilo‐voltage x‐ray images of the BB was acquired and automatic 2D‐to‐3D image registration was performed. The WL QA results were considered acceptable if the 5 mm diameter mechanical BB was conformally encompassed by the 7.5 mm radiation field for every gantry, couch, and collimator angle. On a single strip of Gafchromic film, eight static fields (5 mm BB, with 7.5 mm cone size) with following gantry and couch angles were shot for daily WL QA test (G0, G90, G180, G270; with Couch 0) and (C270, C315, C45, C90; with Gantry 0), respectively. A total of 700 MU/beam was used for WL QA. On each shot, submillimeter coincidence of radiation and mechanical isocenters was maintained at all the times with the use of daily WL QA. In addition to the WL QA, a daily QA check of kilovoltage to megavoltage imaging isocenter coincidence was performed prior to patient setup for TN SRS. All QA procedures were in compliance for radiosurgery treatment delivery including QA for frame placement verification using the depth Helmet bobble measurement.^20^ It was ensured that the originality of the frame placement before CT simulation and prior to treatment was within ± 1 mm of reproducibility.

For each treatment delivery, patient repositioning was achieved using the Target positioner (TaPo) prints out for isocenter localization with the help of gantry cross‐hair. Microadjustments to the couch mount were made following each change in table angle under the supervision of an experienced medical physicist in order to ensure precise isocentricity of each gantry arc. These microadjustment screws on the couch mount allow us to obtain fine adjustment on the TaPo localization in all three directions (anterior, patient left, and right lateral) as well as rotations. Prior to treatment, onboard cone beam CT imaging was performed to verify stereotactic frame placement, head position, and final isocenter location. From the verification cone beam CT, the isocenter localization errors in the left/right, posterior/anterior, and superior/inferior directions were, on average, 0.1 ± 0.7 mm (ranged, −1.0–1.0 mm), 0.3 ± 0.6 mm (ranged, −1.0–1.0 mm), and 0.3 ± 0.8 mm (ranged, −1.0–1.0 mm), respectively. The mean value of angular couch correction discrepancy was 0.1 ± 0.4° (ranged, −0.7–0.6°). These couch correction discrepancies, however, were not applied for the actual treatment considering that these errors were within the range of uncertainty for CBCT image reconstruction and OBI gantry rotation (within ± 1 mm for translational and ± 0.7° for rotational shifts). Overall purpose of verification CBCT was to conform that if there was any unanticipated huge shifts (≥± 2 mm/2°) have been observed, therefore, patient setup could be reconsidered.

### 2.F Patient inclusion, clinical outcome, and toxicity evaluation

For this review, we included a total of 27 refractory TN (*typical* and *atypical*) patients treated at our single institution between 2009 and 2016. All patients reported here were treated by one radiation oncologist and one neurosurgeon. Clinical response to treatment for all patients was retrospectively evaluated and characterized using the Barrow Neurological Institute (BNI) pain intensity score of I–V (see Table 1 for detailed description). At each follow‐up visit, patient‐reported clinical outcomes including use of medical therapy, pain relief, and pain frequency was assessed and incorporated to generate patient respective BNI pain intensity scores. Treatment‐related brainstem or temporal lobe toxicity was evaluated by assessing any clinical symptoms of headache, new cranial nerve deficit, new focal neurological deficit, or presence of seizure activity. If available, temporal lobe necrosis was assessed radiographically by follow‐up MRI brain.

**Table 1:** BNI pain intensity score

Score description	
I	No trigeminal pain, no medications
II	Occasional trigeminal pain that is well tolerated, no medications
III (A–B)	Occasional trigeminal pain that requires medications to be controlled
IV	Some pain that is not adequately controlled with medications
V	Severe pain/no relief

## 3 Results

### 3.A Patient characteristics

The detailed descriptions of patient characteristics are listed in Table 2. Of the 27 refractory TN patients, 22 (81%) suffered from idiopathic/*typical* TN, while 5 (19%) suffered from secondary/*atypical* TN. Median age was 77 yr (ranged, 46–93 yr). Right to left TN ratio was 18/9. Male to female ratio was 14/13.

**Table 2:** Characteristics of 27 clinically followed patients who underwent Linac‐based SRS for refractory trigeminal neuralgia

Characteristics	No. of patients (%)
No. of patients	27
Age (years)	
Median	77
Range	46–93
Gender	
Male	14 (52)
Female	13 (48)
Pain type	
Idiopathic/*Typical* TN (type 1)	22 (81)
Secondary/*Atypical* TN (type 2)	5 (19)
Side	
Right	18 (67)
Left	9 (33)

### 3.B Dosimetric and treatment delivery parameters

**Figure 4:**
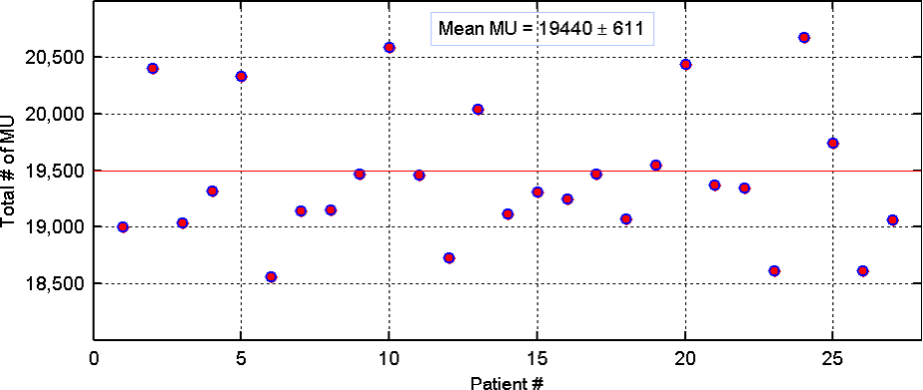
Delivered total number of MU, on a per‐patient basis, for all 27 patients: For 80 Gy prescription dose, the mean value of MU was 19440 ± 611 (ranged, 18,564–20,682).

On a per‐patient basis, the total number of delivered MU for all 27 patients who underwent TN SRS is shown in Fig. 4. In our experience, the mean MU was 19,500 and was fairly standard for all TN patients treated with 80 Gy prescription doses. Knowledge of the average total number of MU is advantageous in that it allows for quick identification of some major errors related to dose calculation — as would be suggested by a calculated total MU which is well above or below the average value.

**Figure 5:**
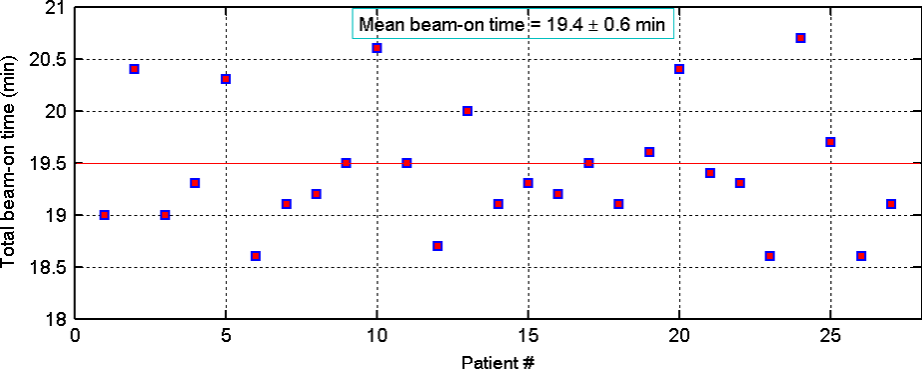
Total beam‐on‐time, on a per‐patient basis, for all 27 patients: mean value of total beam‐on‐time was 19.4 ± 0.6 min (ranged, 18.6–20.7 min) for 80 Gy prescription dose.

In Fig. 5, we show total beam‐on‐time for all 27 patients included in the study. Our average beam‐on‐time was less than 20 min. Understandably, shorter beam‐on‐time helped for patient comfort and faster delivery.

**Figure 6:**
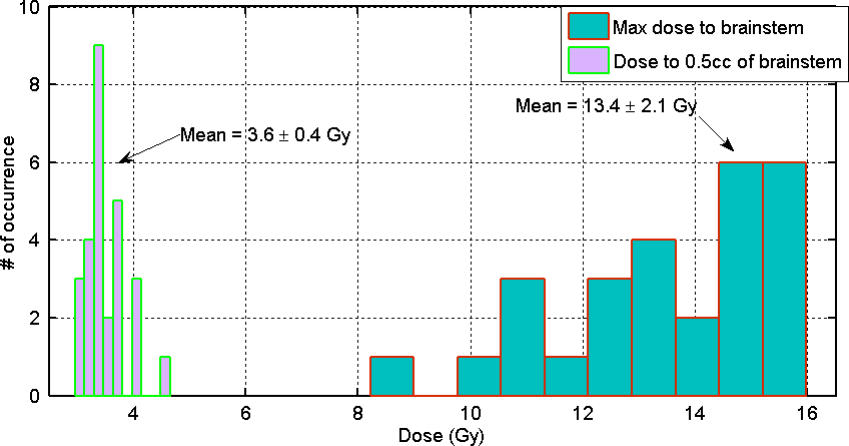
Summary of dose to brainstem (maximum point dose and dose to 0.5 cc of brainstem) for all 27 TNR patients treated with TNR SRS.

Figure 6 demonstrates the ability of linac‐based TN SRS to generate optimal clinical treatment plans that minimize dose to the brainstem. The plot on the left illustrates the 0.5 cc brainstem dose distribution (mean value = 3.6 ± 0.4 Gy, ranged, 1.2–4.8 Gy), and the plot on the right illustrates the maximum brainstem dose distribution (mean value = 13.4 ± 2.1 Gy, ranged, 9.4–15.9 Gy). None of the patients in this study demonstrated evidence of cranial nerve deficit or radio‐necrosis of temporal lobe. In addition, due to the use of orbital avoidance arc arrangement, the maximum dose to optic apparatus was effectively minimized (average <1.2 Gy). Average max‐dose to eyes and lens was 0.3 Gy and 0.2 Gy, respectively.

### 3.C Clinical follow‐up outcomes

**Figure 7:**
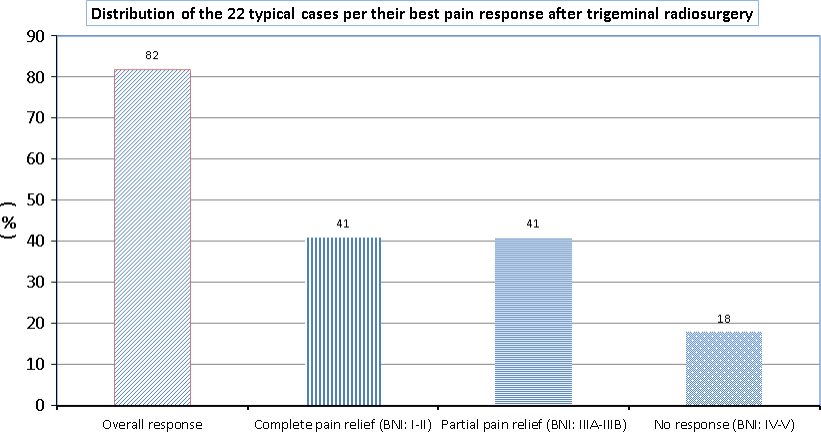
Distribution of the clinical outcomes for only *typical* patients (22/27) treated with TN radiosurgery.

**Figure 8:**
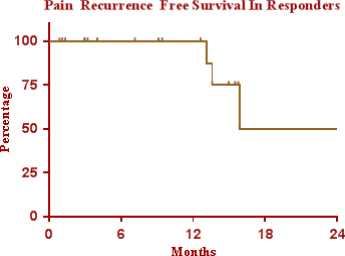
Kaplan–Meier generated curve for actuarial pain recurrence free survival in trigeminal patient responders (BNI: I–IIIB) over time, 22 *typical* TN patients. Actuarial pain recurrence free survival rates were achieved approximately 100%, 75%, and 50% at 12 months, 15 months, and 24 months, respectively.

Median overall follow‐up time was 12.5 months (range 1–53 months). Figure 7 depicts patient‐reported pain relief (in terms of change in the BNI pain intensity score) following SRS for the 22 patients treated for *typical* TN. With a median follow‐up of 12.5 months (ranged, 1–45 months) in this subpopulation, 82% of patients responded to treatment (BNI score I–IIIB). Nine patients (41%) achieved complete pain relief with a BNI score of I–II. Another nine patients (41%) showed partial pain reduction with a BNI score of IIIA–IIIB. Four patients (18%) had no response to radiosurgery treatment — all four having baseline pain of IV–V on BNI scale. For the patients who achieved a response to treatment, the average time to response was 5.5 months (range, immediate to 12 months), and the average duration of response was 13 months (range, 1–53 months). Of the patients who responded to treatment, actuarial pain recurrence free survival rates were approximately 100%, 75%, and 50% at 12 months, 15 months, and 24 months, respectively (see Fig. 8). These results were generated using the Kaplan–Meier product limit method using SPSS 13.0 statistical software (SPSS Inc., Chicago, IL, USA).

While excellent response rates were observed in the patients treated for *typical* TN, none of the patients treated for *atypical* TN responded to treatment. Subset analysis of these five *atypical* patients suggested that 3 (60%) patients had some pain that was not adequately controlled with medications (BNI score IV), while 2 (40%) patients had severe pain without any relief at all (BNI score V). Clinically, the major reasons why there was no response to those five patients who underwent for *atypical* TN needs further investigations.

## 4 Discussion

Using a seven‐arc orbital avoidance arrangement with a 4 mm circular cone size, the maximum dose to the point target was delivered 80 Gy and maximum dose to brainstem never exceeded 16 Gy (20% IDL). At a median follow‐up of 12.5 months, 82% of patients treated for *typical* TN had responded to treatment. Nine patients (41%) had complete pain relief with a BNI score of I–II, while another nine patients (41%) had partial pain relief with a BNI score of IIIA–IIIB. Four patients had no response to radiosurgery treatment, showing that all four patients having baseline pain of IV–V on BNI scale. Actuarial pain recurrence free survival rates for the 22 *typical* TN patients were approximately 100%, 75%, and 50% at 12 months, 15 months and 24 months, respectively. On the other hand, none of the five *atypical* TN patients who underwent linac‐based SRS treatment responded to treatment (BNI score of IV–V).

The safety, efficacy, and localization accuracy of linac‐based TN SRS has been studied by several researchers.^10^, ^11^, ^12^, ^13^ In our clinical implementation of linac‐based TN SRS, we adhered with those standard clinical protocols and guidelines. Treatment planning procedures and patient outcomes for linac‐based TN SRS has also been reported by many investigators.^14^, ^15^, ^16^, ^17^, ^18^, ^19^ For instance, using a seven‐arc geometry with a 4 mm circular cone size, Richards et al.^17^ have shown that overall 75% patient achieved complete pain relief. In their study, 26 patients with medication refractory idiopathic trigeminal neuralgia were treated using an 80 Gy prescription dose and the median follow‐up time was 12 months. In another study with 179 patients, Smith et al.^19^ reported dose‐dependent pain control rates using a 90 Gy prescription dose and a 5 mm cone size. In that study, almost 80% of patients had experienced significant pain relief at a median follow‐up of 28.8 months. Despite the excellent/good pain relief results, the 90 Gy prescription dose and 5 mm cone size resulted in more toxicity with almost 49% of patients developing numbness.

Utilization of CyberKnife SRS for the treatment of TN has also been reported by many investigators.^21^, ^22^, ^23^ Although the treatment outcomes were similar to linac‐based SRS treatment, the reported beam‐on‐times for CyberKnife treatment were relatively longer (~45–60 min) compared to the average beam‐on‐time in our study (~20 min). Our prescription dose was 80 Gy to all patients. By identifying the optimal arc arrangement with a 4 mm circular cone size and differential arc weighting, the dose to the brainstem was minimized such that the average maximum brainstem point dose was less than 16 Gy (20% IDL). There was no posttreatment toxicity such as cranial nerve deficit, numbness, or brain necrosis observed in our study.

In summary, we have presented our faster and robust technique for the treatment of TN utilizing linac‐based SRS. Our overall clinical outcomes suggest that this technique is both safe (less radiation‐induced toxicity) and effective for patients with *typical* TN SRS treatment. However, the lack of success rates for those patients who underwent for *atypical* TN SRS (similar results were presented by Smith et al.^19^) needs further investigations. Due to the advanced of image‐guidance system, linac‐based TN SRS with frameless radiosurgery setup^24^, ^25^, ^26^ merits further investigation.

## 5 Conclusion

In this paper, we have presented our linac‐based SRS treatment procedure for TN and the corresponding clinical outcomes. Our overall response rate was 82% in patients with *typical* TN with half of those patients achieving complete pain relief. Of the patients who responded to treatment, actuarial pain recurrence free survival rates were approximately 100%, 75%, and 50% at 12 months, 15 months, and 24 months, respectively. None of the *atypical* TN patients included in this study had a response to treatment. However, there was no treatment‐related neurological toxicity observed in this study. Longer follow‐up of these patients is anticipated to confirm our clinical observations.

## Conflict of Interest

No conflict of interest.
